# PEDF is an antifibrosis factor that inhibits the activation of fibroblasts in a bleomycin-induced pulmonary fibrosis rat model

**DOI:** 10.1186/s12931-022-02027-4

**Published:** 2022-04-22

**Authors:** Xichun Qin, Caili Jia, Jingtian Liang, Jiali Chen, Xiucheng Liu, Zhixiang Chao, Hao Qin, Yanliang Yuan, Zhiwei Liu, Zhongming Zhang, Hongyan Dong, Hao Zhang

**Affiliations:** 1 Department of Thoracic Surgery, Affiliated Hospital of Xuzhou Medical University, 99 West Huaihai Road, Xuzhou, 221006 Jiangsu China; 2 Morphological Research Experiment Center, Xuzhou Medical University, 209 Tongshan Road, Xuzhou, 221004 Jiangsu China; 3 Department of Thoracic and Cardiovascular Surgery, The Affiliated Drum Tower Hospital of Nanjing University Medical School, Nanjing, 210008 China; 4 Public Experimental Research Center, Xuzhou Medical University, Xuzhou, 221006 Jiangsu China

**Keywords:** PEDF, IPF, Bleomycin, Fibroblast, PPAR-γ

## Abstract

**Background:**

Idiopathic pulmonary fibrosis (IPF) is a highly heterogeneous and fatal lung disease. In addition to dense fibrous tissue, abnormal angiogenesis is also an important feature of IPF. Pigment epithelium-derived factor (PEDF) is an angiogenesis inhibitor and a potential anti-fibrous factor. The purpose of this experiment is to observe the effect of PEDF on bleomycin (BLM)-induced pulmonary fibrosis in rats.

**Methods:**

In vivo, pathological examination and detection of related factors were performed on pulmonary fibrosis induced by BLM in rats, and the temporal and spatial distribution of PEDF was investigated. Furthermore, lung gene delivery (PEDF-adeno-associated virus) was performed to investigate the effect of PEDF on pulmonary fibrosis. In vitro, lentiviral vectors were used to construct PEDF over-expression or knock out primary rat lung (PRL) fibroblasts. The effect of PEDF on fibroblast activation under TGF-β1 stimulation was evaluated, and the activation of TGF-β1/smad pathway and PPAR-γ expression (in the presence or absence of PPAR-γ inhibitors) were analyzed.

**Results:**

In vivo results showed that PEDF expression decreased during the inflammatory phase and increased during the fibrotic phase. PEDF could inhibit the progression of pulmonary fibrosis in rats. In vitro results showed that PEDF could effectively inhibit TGF-β1-stimulated fibroblast activation and reduce the production of α-SMA and collagen-I. PEDF could inhibit the TGF-β1/smad pathway by up-regulating the activity of PPAR-γ.

**Conclusions:**

PEDF can act as an anti-fibrotic factor, inhibit fibroblast activation by upregulating PPAR-γ activity and reduce BLM-induced pulmonary fibrosis in rats.

**Supplementary Information:**

The online version contains supplementary material available at 10.1186/s12931-022-02027-4.

## Background

Idiopathic pulmonary fibrosis (IPF) is the most common interstitial lung disease and one of the most serious diseases of the respiratory system [1, 2]. Factors such as viral infection, radiotherapy/exposure to chemotherapy drugs, and atomized environmental toxins can all cause pulmonary fibrosis [3]. The course of IPF is generally progressive and eventually leads to the death of patients with respiratory failure, affecting more than 5 million people worldwide [4].

Inflammatory damage of lung tissue is one of the main pathological changes in the early stage of pulmonary fibrosis. Severe damage or a repetitive or unbalanced healing response can lead to continuous fibrotic reactions and cause irreversible damage to the lung structure [5, 6]. TGF-β1 is a major pro-fibrosis factor, and its mediated fibroblast activation and myofibroblast accumulation can lead to aggravation of fibrosis, which leads to organ failure [7]. Abnormal angiogenesis is also related to the development of pulmonary fibrosis, and abnormal vascular remodeling in the middle and late stages is a significant feature of pulmonary fibrosis [8]. Past studies have shown that angiogenesis in a fibrotic lung has complex temporal and spatial heterogeneity [9]. The unbalanced expression of angiogenesis factors and angiogenesis inhibitors may cause angiogenesis heterogeneity and patchy results [10].

Pigment epithelial-derived factor (PEDF) is an endogenous glycoprotein originally identified as a neurotrophic factor in retinal pigment epithelial cells [11]. Now, PEDF has been proven to play a central role in mediating cell protection against oxidative stress by promoting cell survival, reducing inflammation, and inhibiting pathological angiogenesis in a variety of cell types and tissues [12]. Previous studies have found that angiogenic factors such as VEGF are reduced in IPF lungs, while the expression of angiogenesis inhibitory molecules (such as PEDF) increased. The expression of PEDF is regulated by TGF-β1, and its expression and distribution in fibrotic areas are relatively increased, suggesting that it may have a potential pathogenic effect [13]. In sharp contrast, in other substantial organ fibrotic lesions, such as kidney [14], heart [15], liver [16], and pancreas [17], PEDF is considered to have an anti-fibrotic effect. Our previous studies revealed that PEDF could reduce β-catenin nuclear translocation and inhibit endothelial–mesenchymal transition in infarcted myocardium [18]. It is worth considering that this seemingly contradictory phenomenon leads to a kind of thinking: whether the abnormal expression and distribution of PEDF is the cause of fibrosis or the result of fibrosis. To date, the specific role of PEDF in pulmonary fibrosis is still unknown.

The microenvironment of damaged tissues usually affects the expression of PEDF [19]. In addition, a characteristic of PEDF is binding the extracellular matrix, such as collagen-I and glycosaminoglycans binding sites, which may be an important reason for its localization in the fibrotic scar area [20]. Although PEDF is currently the most potent anti-angiogenic factor known, it is interesting that PEDF usually targets immature new blood vessels while protecting the mature vasculature [21]. The comprehensive evidence of PEDF function supports its use as a multifunctional regulator of wound healing. In the normal response to injury and the development of fibrotic diseases, PEDF can promote the restoration of damaged tissues to homeostasis [19, 22]. Therefore, the expression of PEDF may be important for the repair of lung tissue. We hypothesize that the up-regulation of PEDF represents a compensation mechanism that inhibits the progression of fibrosis and is necessary to delay the progression of fibrosis.

## Materials and methods

### Rat

Sprague-Dawley (SD) male rats (250 ± 20 g, 8–10 weeks) were obtained from the Experimental Animal Center of Xuzhou Medical College. The rats were kept on a 12 h light–dark cycle with free access to food and water. All experiments were performed in adherence with the National Institutes of Health (NIH Publication, 8th Edition, 2011) guidelines for the use of laboratory animals. The rat care and experimental protocols were examined and verified by Laboratory Animal Ethics Committee of Xuzhou Medical University in accordance with Guide to Laboratory Animal Ethics Examination of Xuzhou Medical University by the Animal Care and Use Committee of Xuzhou Medical University (Ethics Number: 202004B013).

### Reagents and antibodies

The BCA protein concentration determination kit was purchased from Beyotime (Shanghai, China). Tissue or cell total protein extraction kit was purchased from Sangon Biotech (Shanghai, China). TGF-β1, VEGF, TNF-α, IL-1β, and IL-6 ELISA kits were purchased from Shanghai Renjie Biotechnology Co., Ltd. (Shanghai, China). PEDF ELISA kit was purchased from Shanghai Yan Qi Biological Technology Co Ltd. (Shanghai, China). Anti-rabbit serpinf1 (PEDF) antibody (Catalog No. DF6547) was purchased from Affinity Biosciences (Changzhou, Jiangsu, China). Anti-mouse vimentin (Catalog No. ab8978), anti-rabbit von Willebrand factor (Catalog No. ab154193), anti-rabbit factor VIII (Catalog No. ab236284), Anti-mouse smooth muscle actin (Catalog No. ab7817), anti-rabbit PPAR-γ (Catalog No. ab178860) antibodies, and PPAR-γ inhibitor (GW 9662, Catalog No. ab141125) were purchased from Abcam (Cambridge, MA, USA). Anti-rabbit smooth muscle actin (Catalog No. 14395-1-AP), anti-rabbit collagen type I (Catalog No. 14695-1-AP), anti-mouse β-tubulin (Catalog No. 66240-1-Ig) antibodies, and animal-free recombinant human TGF-β1 (Catalog No. Cat No. HZ-1011) were purchased from Proteintech (Wuhan, Hubei, China). Anti-rabbit phospho-smad2/smad3 antibody (Catalog No. 8828S) was purchased from Cell Signaling Technology (Danvers, MA, USA).

### Adeno-associated virus (AAV) and lentivirus (LV) preparation

Recombinant adeno-associated virus (PEDF-AAV and PEDF-shRNA-AAV) and lentivirus (PEDF-LV and PEDF-shRNA-LV) was prepared by Shanghai Jikai Gene Medical Technology Co., Ltd. [23, 24]. In brief, PEDF overexpression plasmids and the RNAi vector were successfully constructed and then packaged in 293T cells. The concentrated titer of LV suspension was 2 × 10^12^ TU/L and AAV was 2 × 10^14^ TU/L.

### Rat IPF model and gene delivery

SD rats were anesthetized with sodium pentobarbital (60 mg/kg) intraperitoneally and intubated with a 16G intravenous indwelling needle. BLM (5 mg/kg or 2 mg/kg) was instilled intratracheally. The rats were rotated immediately after instillation to ensure thorough drug distribution in the lungs. PEDF-AAV or PEDF-shRNA-AAV (2 × 10^13^ TU) prepared in 200 μL enhanced infection solution delivered by the same method before 3 weeks or 1 day models were made.

### Histologic assessment and hydroxyproline assay of lung tissues

Left lungs were immersed in 4% paraformaldehyde for 24 h, dehydrated, embedded in paraffin, and then the tissues were cut into 4-μm-thick sections and stained with hematoxylin and eosin and Masson’s trichrome. Ashcroft’s degree of fibrosis was performed by two blinded investigators.

Hydroxyproline was measured using the hydroxyproline test kit according to the manufacturer’s instructions. Data were expressed as micrograms of hydroxyproline per gram of wet lung weight.

### Reverse transcription‑quantitative polymerase chain reaction (RT‑qPCR) analysis

The total cardiomyocyte RNA was extracted using TRIzol reagent following the manufacturer’s protocol. The RNA (1000 nmol) was then subjected to reverse transcription with the Prime Script RT reagent kit and gDNA Eraser. PCR was conducted with a final volume of 20 µL containing 10 µL 2× SYBR‑Green PCR Master mix, 0.1 µM of each primer, and 100 µg genomic DNA. The mixture was subjected to qPCR amplification (95 °C for 10 min), 45 cycles (95 °C for 10 s, 60 °C for 10 s, 72 °C for 20 s), and one cycle (95 °C for 1 min, 65 °C for 1 min, and 97 °C with continuous) and then cooled to 40 °C for 30 s using a Roche Light Cycler 480 (Roche Diagnostics GmbH, Mannheim, Germany). Gene expression was normalized to that of 18s RNA. Gene expression was quantified using the 2‑∆∆Cq method. The following primers, synthesized by GenScript (Piscataway, NJ, USA), were used: TGF-β1 forward, 5ʹ‑CTGCTGACCCCCACTGATAC‑3ʹ and reverse, 5ʹ‑AGCCCTGTATTCCGTCTCCT‑3ʹ; VEGF forward, 5ʹ‑AGCCCGGAAGATTAGGGAGTT‑3ʹ and reverse, 5ʹ‑CCAGGGATGGGTTTGTCGTGT‑3ʹ; PEDF forward, 5ʹ‑CAGAGTCTGTCATTCACCGGGC‑3ʹ and reverse, 5ʹ‑GTCAGCACAGCTTGGATAGTCTTC‑3ʹ. 18sRNA forward, 5ʹ‑CCTGGATACCGCAGCTAGGA‑3ʹ and reverse, 5ʹ‑GCGGCGCAATACGAATGCCCC‑3ʹ. The levels of mRNA were quantified through 18S rRNA to normalize TGF-β1, VEGF and PEDF.

### Immunofluorescence staining

Sections were deparaffinized and dehydrated, immersed in 10 mM sodium citrate (pH 6.0) solution, microwave on high heat for 5–10 min for antigen retrieval. Then, sections were permeabilized with Triton X-100 (0.1%) and blocked with a solution containing 5% bovine serum before applying the primary antibody. Specimens were incubated with anti-mouse SMA antibody and anti-rabbit PEDF antibody for 12 h at 4 °C and incubated with secondary antibodies under light-protected conditions for 1 h at room temperature. Nuclei were stained with DAPI. After a final washing, coverslips were mounted on the slides using 50% glycerin. Then, the sections were observed under a fluorescence microscope (Olympus).

### Immunohistochemical staining (IHC)

After antigen retrieval, the lung tissue sections were blocked with goat serum for 20 min then were incubated with anti-CD34 for 12 h at 4 °C. Next, the sections were washed twice with PBS and subsequently incubated with HRP polymer-conjugated secondary antibody at room temperature for 15–30 min. Finally, the sections were stained with hematoxylin and eosin. The slides were photographed with an inverted microscope (Olympus).

### Quantification of cytokines and protein permeability index (PPI)

Bronchoalveolar lavage fluid (BALF) was performed on the lungs using an intravenous indwelling needle with 5 mL of cold PBS (pH 7.4). Lung tissues (100 mg) from normal and IPF rats were homogenized with 1 mL of cold PBS (pH 7.4). The supernatant of BALF and tissue homogenate was collected by centrifugation (5000*g*, 5 min). TGF-β1, VEGF, PEDF, TNF-α, IL-1β, and IL-6 levels were measured using quantitative ELISA kits according to the manufacturer’s protocols. The protein permeability index was calculated as BALF total protein/plasma total protein × 100.

### Isolation of primary rat lung (PRL) fibroblasts

The rats were sacrificed by cervical dislocation, fixed, the chest skin sliced, and the lung isolated. An intravenous indwelling needle was inserted into the trachea, and the lung tissue was repeatedly cut into tissue pieces with a size of 1 mm × 1 mm and planted in a gelatin-coated culture flask. 20–25 pieces per bottle, 0.5 cm apart. 2 mL of MEM medium containing 10% fetal bovine serum was added, and the culture flask was placed in an incubator obliquely. When it stuck to the bottom for 2–4 h, the culture flask was slowly turned over and laid flat so that the culture medium could infiltrate the tissue mass. After 72 h, many PRL fibroblasts could be seen crawling out under the microscope. The tissue masses were then removed, and the culture and passage continued.

### Cell culture and treatment

PRL fibroblasts were cultured in MEM culture medium containing 10% fetal bovine serum and incubated in a humidified atmosphere containing 5% CO_2_. TGF-β1 (10 ng/mL) was added for 48 h to induce activation of PRL fibroblasts. LVs gene delivery was based on the number of cells (multiplicity of infection = 20). At the corresponding time point, total protein was extracted according to the manufacturer’s instructions.

### Western blot analysis

Proteins from lung tissues and PRL fibroblasts were extracted using a cell or tissue total protein extraction kit. A BCA protein concentration determination kit was used to determine the protein concentration of each sample. Proteins were separated by SDS-PAGE and transferred onto nitrocellulose membranes. After blocking in 5% nonfat milk for 2 h, the membranes were incubated with primary antibodies against PEDF, α-SMA, collagen-I, PPAR-γ, phospho-smad2/smad3, or β-tubulin overnight at 4 °C. After washing, the membranes were incubated with fluorescently labeled anti-mouse or anti-rabbit secondary antibodies at room temperature for 1–2 h, and the blot was then imaged using the Odyssey infrared imaging system (Li-Cor). Densitometric analysis of the bands was performed using ImageJ software. Protein levels were calculated from the ratio of corresponding protein/β-tubulin.

### Statistical analysis

Data were expressed as mean ± standard deviation (SD). Multiple group comparisons were evaluated via one-way ANOVA followed by least significant difference t-test for post hoc analysis. Data between two independent groups were compared using a two-tailed Student’s t-test. Analyses were performed using SPSS 25 software (Chicago, IL, USA). Differences with P < 0.05 were considered statistically significant.

## Results

### Characterization of bleomycin-induced pulmonary fibrosis in rats

**Fig. 1:**
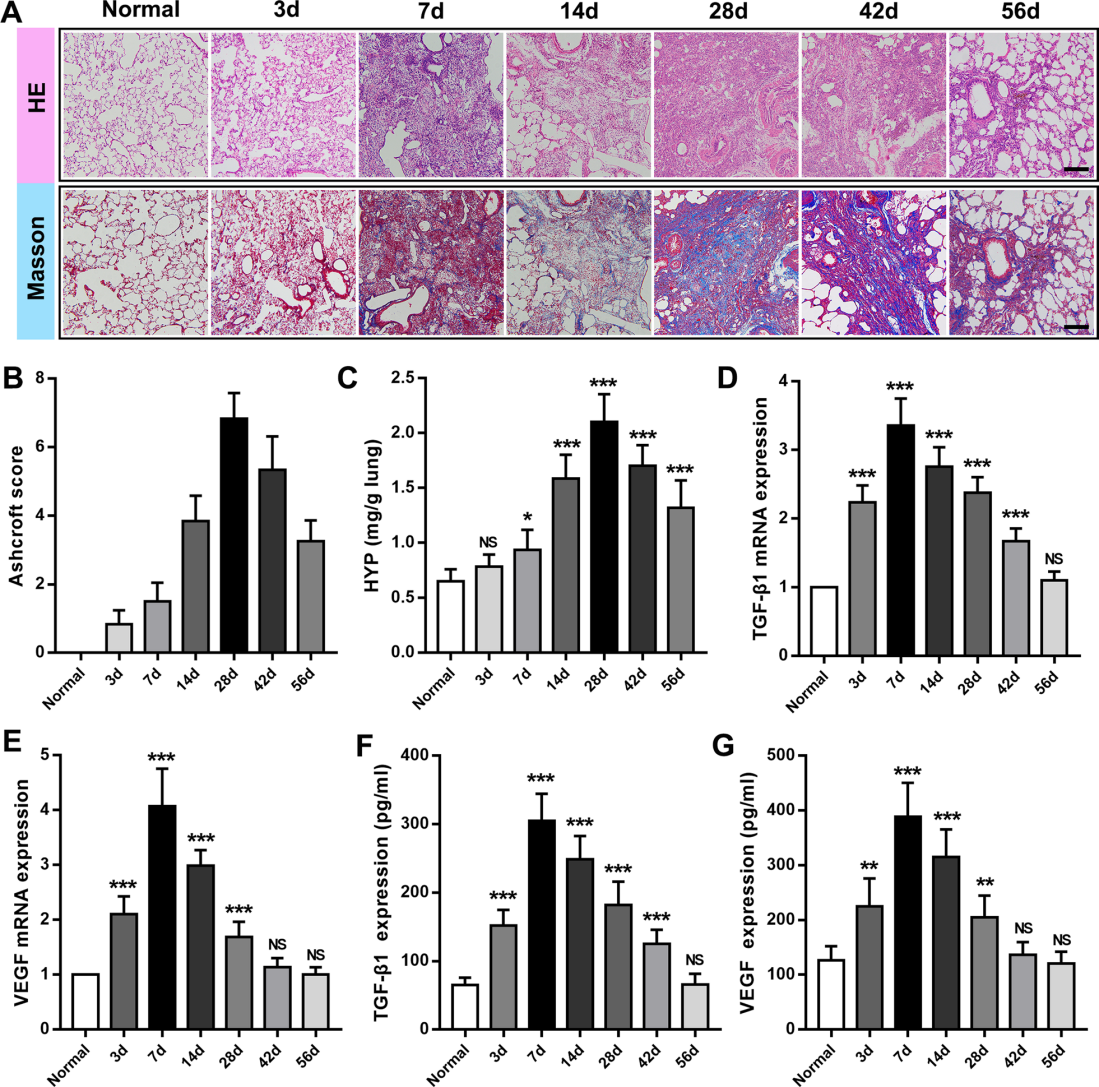
Progression characteristics of BLM-induced IPF. **A** H&E and Masson-stained cross-section of the lung from normal and BLM-exposed rats (3 days, 7 days, 14 days, 21 days, 28 days, 42 days, 56 days). Bar = 200 μm. **C** The Ashcroft score among multiple groups. **D** Quantification of hydroxyproline content in lung tissue. **E** TGF-β1 mRNA and **F** VEGF mRNA expression was measured by real-time PCR. **G** TGF-β1 and **H** VEGF content of lung tissue from multiple groups. Data are expressed as the mean ± SD, n = 6, experimental group vs normal group, NS, no significant difference; *P < 0.05; **P < 0.01; ***P < 0.001

To observe the dynamic changes of pulmonary fibrosis in detail, we investigated the pathological characteristics of bleomycin-induced pulmonary fibrosis in rats within 2 months. H&E and Masson staining were used to observe the morphological changes of lung tissue. The Ashcroft score was used [25], and the deposition of hydroxyproline in lung tissue was detected to assess the degree of pulmonary fibrosis. We found that in the early stage of BLM treatment, the lung tissue mainly manifested as alveolar septum widening and inflammatory cell infiltration. On the 14th day, it transitioned to nodular changes, with a significant increase in fibroblasts and more fibrous tissue proliferation. On the 28th day, the fibrous foci were the most serious (Fig. 1A), and during this period, Ashcroft score (6.83 ± 0.57) and hydroxyproline deposition (2.10 ± 0.25 mg/g) also reached their peaks (Fig. 1B and C). In the following 4 weeks, the degree of fibrosis was reduced, indicating that the pathological changes in fibrosis induced by BLM were naturally reversed (Fig. 1A–C).

TGF-β1 is a key mediator of fibrosis in many organ systems, especially in IPF [26]. VEGF is also considered to be an important factor in promoting fibrosis [8, 27]. We found that the expression changes of TGF-β1 are similar to VEGF by RT-qPCR method. Both increased significantly in the early stage of injury, reaching a peak at 7 days (mRNA 3.35 ± 0.40 vs. 1.00; 4.07 ± 0.69 vs. 1.00), and continued to be highly expressed after the 14th day, then gradually decreased, reaching the normal level at 56 days (mRNA 1.10 ± 0.14 vs. 1.00; 1.01 ± 0.13 vs. 1.00). Their quantitative results also supported the above findings (Fig. 1F and G). It was worth noting that there were still significant fibrotic lesions in the lung tissue during this period. TNF-α, IL-1β, and IL-6 are classic inflammatory factors, which are commonly used to reflect the level of inflammation in lung tissue [28]. Quantitative results of the concentration of TNF-α, IL-1β, and IL-6 inflammatory factors in BALF (Additional file 1: Fig. S1A–C) showed that the peak of the expression of each inflammatory factor occurred on the 3rd day (274 ± 28 pg/mL; 75 ± 10 pg/mL; 234 ± 25 pg/mL), and then gradually decreased, returning to normal levels at 42 days (103 ± 14 pg/mL vs. 106 ± 18 pg/mL; 29 ± 5 pg/mL vs. 26 ± 7 pg/mL; 96 ± 12 pg/mL vs. 94 ± 11 pg/mL).

### Angiogenesis and the temporal and spatial distribution of PEDF

**Fig. 2:**
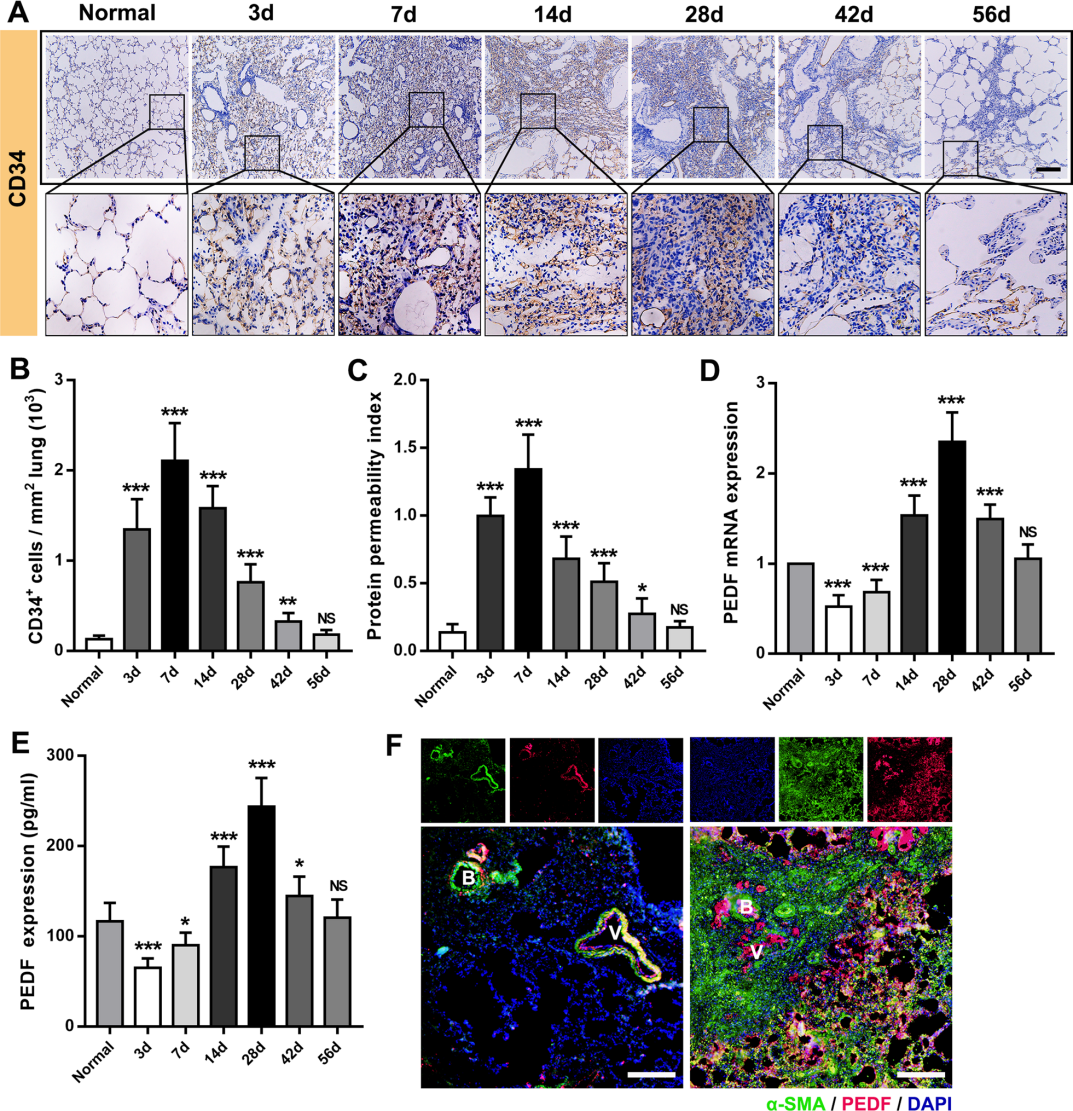
Angiogenesis and PEDF expression and distribution. **A** Immunohistochemical staining of CD34 in lung sections. Bar = 200 μm; **B** Quantification of cells positive for CD34. **C** Representative PPI change, calculated as BALF total protein/plasma total protein × 100. **D** PEDF mRNA expression was measured by real-time PCR. **E** PEDF content of lung tissue. Data are expressed as the mean ± SD, n = 6, experimental group vs normal group, NS, no significant difference; *P < 0.05; **P < 0.01; ***P < 0.001. **F** Immunofluorescent staining showed the localization and expression of PEDF in the lung from normal and BLM-exposed (28 days) rats. Bar = 200 μm

In addition to dense fibrous connective tissue hyperplasia, another typical feature of fibrosis is abnormal angiogenesis, especially in BLM-induced animal lung fibrosis. This period represents the renewal of endothelial cells, and therefore the number of CD34^+^ cells that can initially reflect microangiogenesis has increased significantly [8]. The rat IPF model (Fig. 2A and B) showed that the number of CD34^+^ cells reached a peak on day 7 (2.11 ± 0.42 × 10^3^/mm^2^). In addition, the ratio of total BALF protein/total plasma protein proved that after BLM attack [29], severe leakage of the pulmonary vascular system was caused in the early stage (Fig. 2C). Consistent with the peak period of CD34^+^ cells, vascular leakage was the most serious on day 7 (1.34 ± 0.26), indicating that angiogenesis might be the most significant during this period. PEDF has been confirmed to be expressed in a variety of cells. We observed that in normal rat lung tissue, the expression of PEDF was mainly concentrated in atmospheric ducts and blood vessels with a diameter of 50 μm or more. In fibrotic lung tissue, we found that PEDF expression was enhanced, and it was mainly concentrated in the thickened interstitial area of the lung (Fig. 2F). RT-qPCR results showed that the expression level of PEDF was significantly different from that of TGF-β1 and VEGF. In the early stage of BLM attack (3 days group and 7 days group), the relative mRNA level of PEDF and the expression concentration of lung tissue decreased significantly (Fig. 2D and E). At this time, the lung tissue was in a significant period of inflammation. As time prolonged and the degree of fibrosis deepened, the expression of PEDF showed an upward trend and exceeded the normal level. On the 28th day, the most severe period of fibrosis, the PEDF level reached a peak (mRNA 2.35 ± 0.32 vs. 1.00; 244 ± 32 pg/mL), then began to decline, and finally returned to the baseline level on the 56th day (Fig. 2D and E).

### Adeno-associated virus vector-mediated PEDF overexpression inhibits the progression of pulmonary fibrosis

**Fig. 3:**
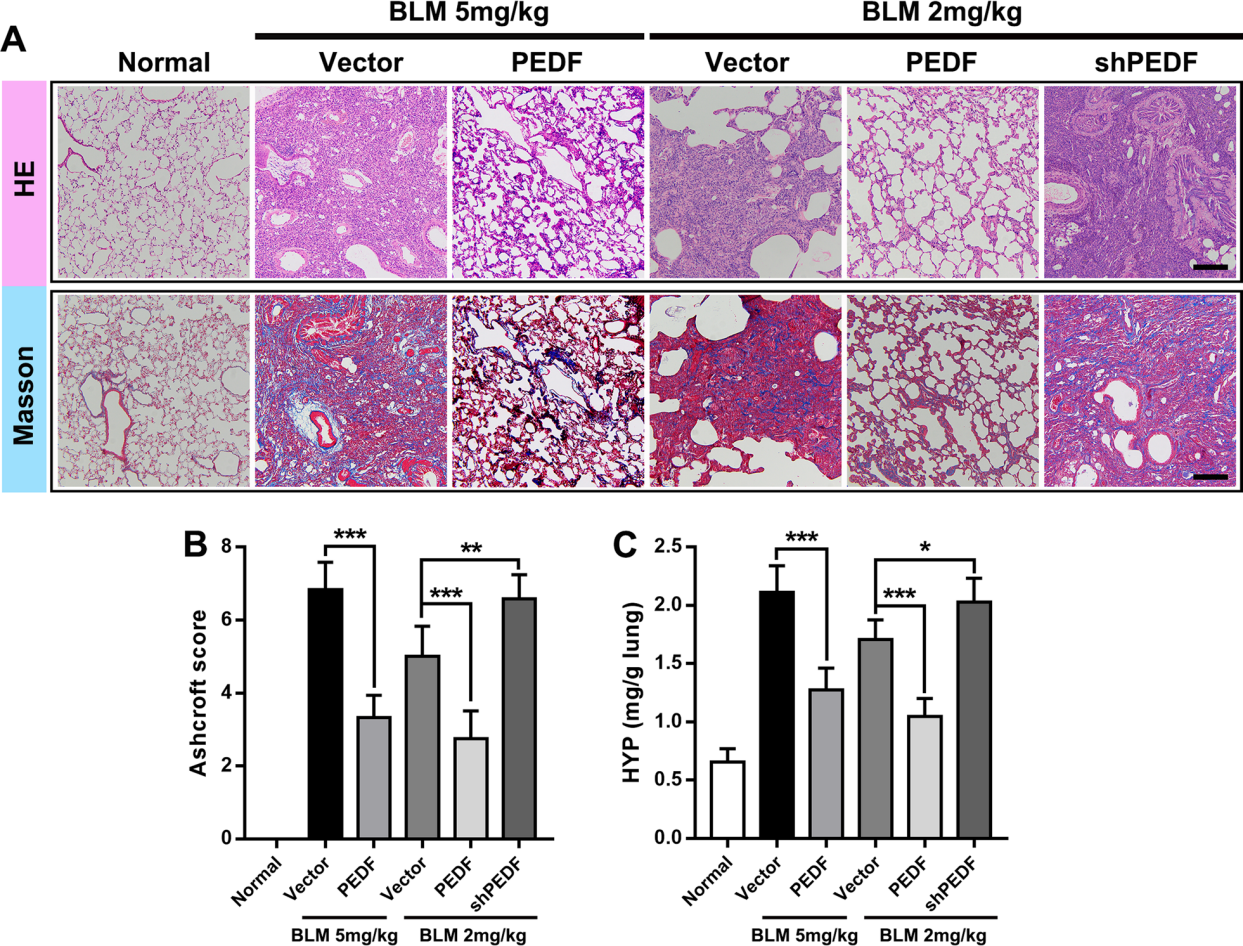
PEDF could inhibit fibrosis induced by BLM. **A** Representative images of the H&E and Masson-stained cross-section of the lung from the experimental group (BLM 5 mg/kg or 2 mg/kg) and PEDF-AAV (denoted as PEDF) treatment group or PEDF-shRNA-AAV (denoted as shPEDF) treatment group. BLM (2 mg/kg or 5 mg/kg) represents lung tissue from rats 28 days after BLM instillation. Bar = 200 μm. **B** The Ashcroft score among multiple groups. **C** Quantification of hydroxyproline content in lung tissue of each group. Data are expressed as the mean ± SD, n = 6, *P < 0.05; **P < 0.01; ***P < 0.001

To determine the specific role of PEDF in the process of pulmonary fibrosis, overexpression or interference with PEDF expression by the adeno-associated virus was analyzed. After tracheal instillation of adeno-associated virus, it began to express weakly after 2 week and expressed more significant transfection efficiency after 3 week (Additional file 1: Fig. S2A–C). For this reason, we chose to instill the AAV virus into the trachea 3 weeks in advance. 5 mg/kg BLM is recommended as the standard measurement for animal models of pulmonary fibrosis [30]. However, we found that the administration of 5 mg/kg BLM almost reached the lethal dose in the shPEDF group (Additional file 1: Fig. S2D). Therefore, we increased the low-dose BLM group to observe the effect of shPEDF on pulmonary fibrosis in rats. And according to the previous results, the sampling time was set as 28 days after BLM instillation to evaluate the biological effect of PEDF. In addition, we also demonstrated that PEDF-AAV mediated higher expression of PEDF in lung tissue in the stage of severe fibrosis (Additional file 1: Fig. S2E and F). As shown in Fig. 3A, BLM at a dose of 2.0 mg/kg still caused significant fibrosis characteristics (Ashcroft score and hydroxyproline deposition in lung tissue increased considerably). We found that the PEDF group substantially inhibited the occurrence of pulmonary fibrosis. On the contrary, the shPEDF group showed a more severe degree of fibrosis (Fig. 3A–C).

It is important to distinguish between drugs that inhibit the inflammatory response and prevent the progression of fibrosis [4]. It has been reported that PEDF was an endogenous anti-inflammatory factor [31]. Our study also proved that PEDF could significantly inhibit the levels of TNF-α, IL-1β and IL-6 (3 days after BLM instillation) (Additional file 1: Fig. S3A–C). To further clarify the anti-fibrotic properties of PEDF, we observed the therapeutic effect of PEDF in the established fibrosis stage. The AAVs were instilled with a tube on the day before the model was made, and the material was taken 6 weeks after the model was made to evaluate the effect of PEDF’s delayed treatment. The results showed that even with delayed intervention, PEDF could promote the regression of fibrosis, which further proved the anti-fibrotic effect of PEDF (Additional file 1: Fig. S4A–E).

### PEDF inhibits the TGF-β1/smad pathway of fibroblasts and reduces the production of α-SMA and collagen-I

**Fig. 4:**
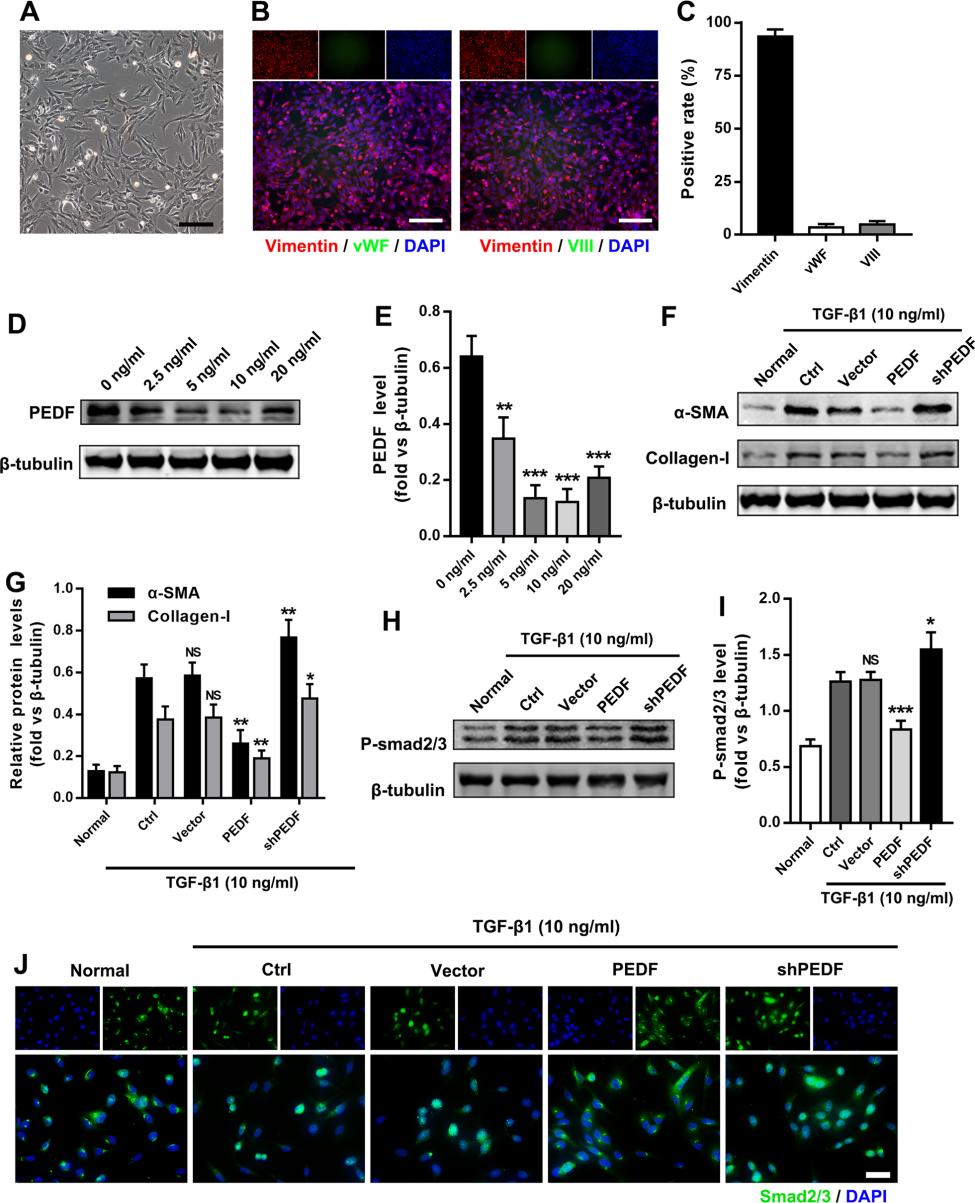
PEDF inhibited fibroblast activation by inhibiting the TGF-β1/smad2/3 pathway. **A** Observation of the morphological characteristics of PRL fibroblasts by microscope. Bar = 50 μm; **B** Identification of PRL fibroblasts using fluorescence microscope. Vimentin positive, von Willebrand factor (vWF), and factor VIII negative cells stand for PRL fibroblasts. Bar = 100 μm. **C** Quantitative statistics of the positive rate of each factor. **D** and **E** Western blot to detect the effect of different concentrations of TGF-β1 on PEDF expression. **F** and **G** Western blot to detect the effect of PEDF on α-SMA and collagen-I expression. **H** and **I** Western blot to detect the effect of PEDF on phospho-smad2/smad3 expression. PEDF-LV (denoted as PEDF) treatment group or PEDF-shRNA-LV (denoted as shPEDF) treatment group vs. Ctrl group. Data are expressed as the mean ± SD, n = 4, NS, no significant difference; *P < 0.05; **P < 0.01; ***P < 0.001. **J** Immunofluorescence to observe the effect of PEDF on the intracellular distribution of smad2/3. Bar = 20 μm

To determine how PEDF affected the activation of fibroblasts, we obtained primary rat lung (PRL) fibroblasts from neonatal rat lung tissue. As shown in Fig. 4A, the PRL fibroblasts were in the shape of a long spindle or flat star under the light microscope. We tested the purity of PRL fibroblasts by immunofluorescence (Fig. 4B and C). The results showed that the vimentin positive rate of PRL fibroblasts was about 95%, while Factor VIII and vWF were negative (both positive rates were less than 5%). Previous studies reported that TGF-β1 up-regulated the expression of PEDF in 3T3-L1 fibroblasts [13]. However, we found that after using different concentrations of TGF-β1 (2.5 ng/mL, 5 ng/mL, 10 ng/mL, 20 ng/mL) to stimulate PRL fibroblasts, the expression of PEDF decreased significantly (Fig. 4D and E). After treatment with PEDF lentivirus (Additional file 1: Fig. S5A and B), the expression levels of α-SMA and collagen-I induced by TGF-β1 decreased significantly; on the contrary, when the expression of PEDF was inhibited, the expression levels of α-SMA and collagen-I in PRL fibroblasts increased significantly (Fig. 4F and G). This supported that PEDF could effectively inhibit the activation of PRL fibroblasts mediated by TGF-β1.

Smad2/3 protein plays a key role in the process of transmitting TGF-β1 signals from cell surface receptors to the nucleus [32], so we measured the effect of PEDF on the activation of smad2/3 induced by TGF-β1. The results showed that after TGF-β1 treatment, the phosphorylation level of smad2/3 in PRL fibroblasts significantly increased. Lentiviral vector-mediated PEDF overexpression effectively blocked the phosphorylation of smad2/3 (Fig. 4H and I). Furthermore, immunofluorescence results also showed that PEDF could inhibit the smad2/3 nuclear translocation (Fig. 4J). These results indicated that the anti-fibrotic effect of PEDF might be achieved by interfering with the TGF-β1/smad signaling pathway.

### PEDF inhibits TGF-β1/smad pathway by up-regulating PPAR-γ activity

**Fig. 5:**
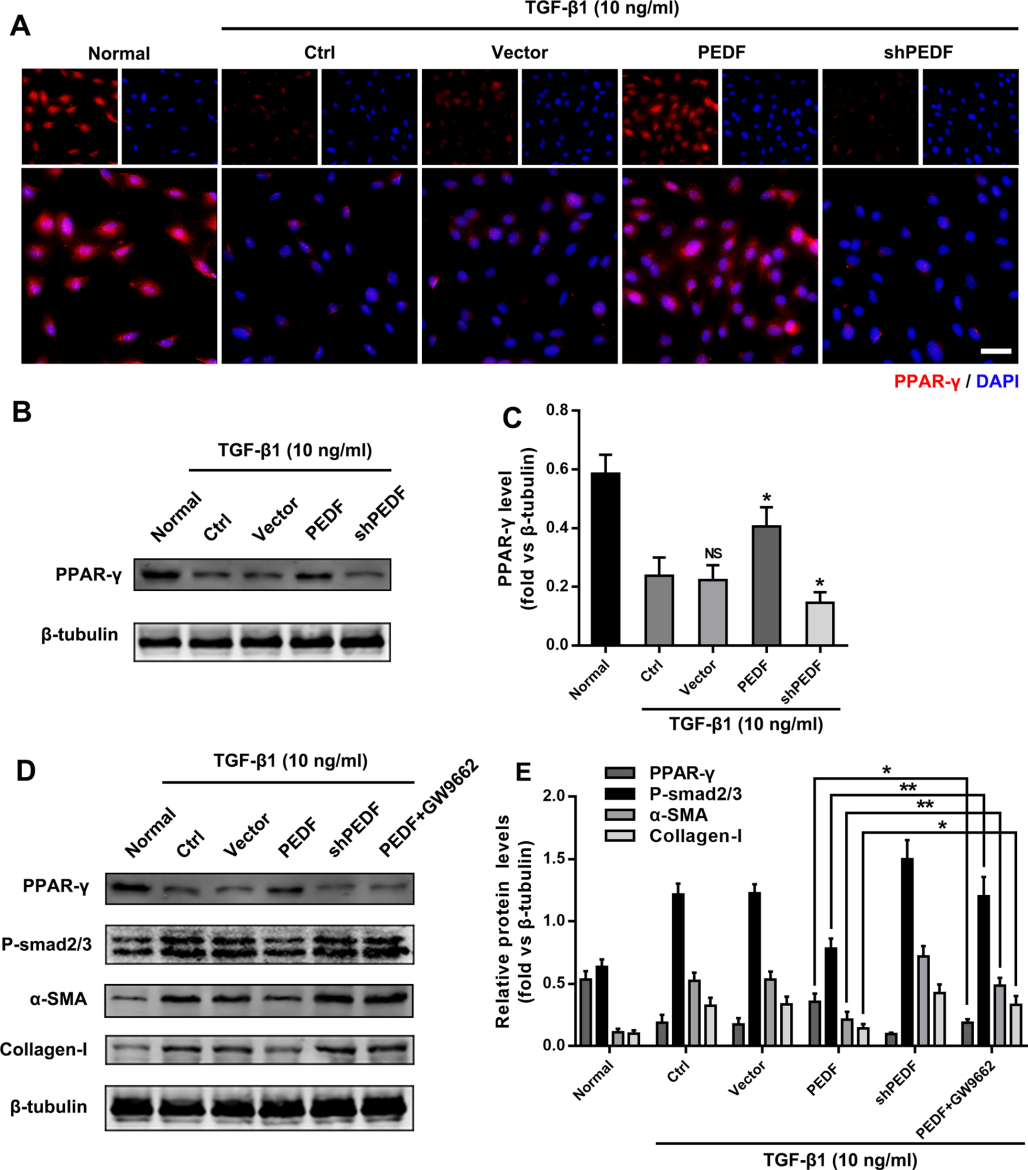
PEDF inhibited fibroblast activation by up-regulating PPAR-γ expression. **A** Immunofluorescence to observe the effect of PEDF on the expression of PPAR-γ. Bar = 20 μm. **B** and **C** Western blotting to detect the effect of PEDF on the expression of PPAR-γ. LV-group vs Ctrl group. **D** Immunoblot analysis of PPAR-γ, phospho-smad2/smad3, α-SMA, and collagen-I. Quantification of the related protein expression showed significant downregulation of phospho-smad2/smad3, α-SMA, and collagen-I, which was reversed by the inhibitor GW 9662. Data are expressed as the mean ± SD, n = 4, NS, no significant difference; *P < 0.05; **P < 0.01

Peroxisome proliferator-activated receptor γ (PPAR-γ) was a transcription factor that could heterodimerize with retinoid X receptors and activate genes involved in lipohomeostasis [33]. Studies have proved that PPAR-γ activity in lung tissues of patients with pulmonary fibrosis was reduced, and PPAR-γ agonists could inhibit the progression of pulmonary fibrosis [34]. Our previous studies have shown that PEDF could up-regulate the activity of PPAR-γ, which was an important mechanism for protecting the survival of ischemic cardiomyocytes [35]. Whether PEDF up-regulated the activity of fibroblast PPAR-γ and affected the process of pulmonary fibrosis remains to be determined. Consistent with our expectations, after receiving TGF-β1 stimulation, the PPAR-γ activity of PRL fibroblasts was reduced significantly, and overexpression or knockdown of PEDF reversed or exacerbated this change (Fig. 5A–C). PRL fibroblasts treated with the inhibitor GW9662 (PPAR-γ ligand binding domain antagonist) reversed the anti-fibrotic effect of PEDF (Fig. 5D and E). In summary, our findings indicated that PEDF inhibited the phosphorylation of smad2/3 by regulating PPAR-γ.

## Discussion

IPF is a major human health problem, and the exact pathogenic mechanism is still unclear. Past studies have suggested that PEDF was a regulator of pulmonary angiogenesis and an important mediator of IPF, but the specific role of PEDF in IPF is still uncertain [13]. We constructed a rat IPF model through tracheal infusion of BLM and set up multiple observation time points to reveal the specific functions of PEDF in the process of IPF rats. We found that PEDF expression exceeded the normal level that occurred in the early stage of fibrosis evolution and reached the peak in the most severe period of fibrosis; this is in line with the findings of past studies. High expression of PEDF has been observed in the tissues of patients with pulmonary fibrosis. In the early stage of injury, the expression of PEDF is lower than normal. The increase in the expression of PEDF is accompanied by the start of lung self-repair, and during this period, as the expression of PEDF increases, the expression of TGF-β1 and VEGF begins to decrease. The administration of PEDF adeno-associated virus accelerated the regression of fibrosis, while shPEDF aggravated the degree of pulmonary fibrosis in the same period, which proved the anti-fibrosis effect of PEDF.

TGF-β1 can regulate the expression of PEDF [13]. We found that the area of high fibrotic activity was also the area of increased PEDF activity, so we further studied the relationship between PEDF and TGF-β1. Our study showed that TGF-β1 did not increase the expression of PEDF in primary rat lung fibroblasts. On the contrary, with the administration of TGF-β1, the expression of PEDF decreased to varying degrees. This finding is contrary to past research, which may be due to studies using different cell types. For example, in human dermal fibroblasts, TGF-β1 could inhibit the expression of PEDF by up-regulating PDGF [36]. We found that PEDF could significantly inhibit the TGF-β1/smad signaling pathway, thereby inhibiting the expression of α-SMA and collagen-I in fibroblasts. It has been observed that the expression of PPAR-γ in the lung tissue of patients with IPF decreased [34]. Similarly, we found that TGF-β1 could inhibit the activity of PPAR-γ in RPL fibroblasts, and with the intervention of PEDF, the activity of PPAR-γ has been restored. PPAR-γ inhibitors reversed the anti-fibrosis effect of PEDF to a certain extent, indicating that PEDF’s inhibition of smad2/3 phosphorylation might be achieved by increasing the expression of PPAR-γ. Since PEDF itself also serves as an endogenous anti-inflammatory factor, to rule out the possibility of anti-inflammatory effects in the early stage of administration for the preventive treatment of pulmonary fibrosis, we examined the effect of delaying PEDF treatment. We found that even if the treatment was delayed, PEDF still accelerated the regression of fibrosis, which further proved the anti-fibrotic effect of PEDF.

Vascular abnormalities in IPF involved all parts of the vascular bed [37]. The BLM-induced IPF used in this study involved inflammation and induced a pro-angiogenic environment in the lung [30]. Based on human studies and animal lung fibrosis induced by bleomycin, the association between excessive angiogenesis and fibrosis in IPF had been proposed [29]. Compared with normal rat lung tissue, we noticed that on the 7th day after BLM challenge, the number of CD34-positive cells was the largest, indicating that angiogenesis might be the most significant during this period. Correspondingly, the overall level of PEDF was lower than normal in the first 7 days, and the expression of VEGF gradually reached its peak. In the early stage of injury, the connection of new blood vessels is usually immature, which may aggravate the occurrence of inflammation in the early stage of injury. This experiment does not include studies on secondary pulmonary hypertension. Vascular inhibitor therapy may lead to the progression of pulmonary hypertension and may also negatively impact pulmonary hemodynamics. PEDF is currently the strongest angiogenic inhibitor known, but it only has a pro-apoptotic effect on neonatal endothelial cells. Interestingly, in the study of rat heart microcirculation, PEDF could activate NO and Notch-1 signaling pathways to reshape natural collaterals. Although it reduced the number of blood vessels, it increased the diameter of blood vessels and ultimately promoted an increase in net blood flow [38, 39]. It is unclear whether this property of PEDF can be applied to the new capillary network in inflammatory injury. However, some studies have pointed out that in the proliferation stage of skin wound healing, after the burst of angiogenesis caused by hypoxia, PEDF helped heal the wound by causing the degeneration of immature blood vessels and stimulating the maturation of the vascular microenvironment [19, 22]. The BLM-induced IPF model is not an ideal IPF animal model, and it does not have the characteristics of slow progress and irreversibility [40]. Over time, the diseased lung tissue can gradually recover [30]. However, the mechanism that promotes the resolution of fibrosis is not well understood. We speculate that the upregulation of PEDF in a fibrotic lung may be a compensatory increase in the self-reducing mechanism.

## Conclusion

Taken together, our research reveals that PEDF has an undiscovered beneficial effect in pulmonary fibrosis; that is, it can inhibit the activation of fibroblasts caused by TGF-β1 by regulating the activity of PPAR-γ, thereby inhibiting the development of fibrosis. The evidence we provided suggested that the biological function of PEDF might be a new strategy for the treatment of progressive fibrotic diseases.

## Supplementary Information

**Additional file 1: Figure S1.** Quantification of cytokines. **Figure S2.** PEDF expression level in rats. **Figure S3.** PEDF could inhibit BLM-induced inflammatory response. **Figure S4.** Delayed intervention of PEDF could also inhibit collagen deposition. **Figure S5.** PEDF expression level in RPL fibroblasts.

## Data Availability

The datasets supporting the conclusions of this article are included within the article.

## Abbreviations

IPF: Idiopathic pulmonary fibrosis
PEDF: Pigment epithelial-derived factor
BLM: Bleomycin
TGF-β1: Transforming growth factor β1
VEGF: Vascular endothelial growth factor
α-SMA: Alpha-smooth muscle actin
TNF-α: Tumor necrosis factor-α
IL-1β: Interleukin-1β
IL-6: Interleukin-6
BALF: Bronchoalveolar lavage fluid
PPAR-γ: Peroxisome proliferator-activated receptor γ
