# Supplementary material for: PEDF is an antifibrosis factor that inhibits the activation of fibroblasts in a bleomycin-induced pulmonary fibrosis rat model
Source: Respir Res. 2022 Apr 22;23:100. doi: 10.1186/s12931-022-02027-4 (PMC9027047; doi:10.1186/s12931-022-02027-4)
Supplement: Supplementary file 1 — Additional file 1: Figure S1. Quantification of cytokines. Figure S2. PEDF expression level in rats. Figure S3. PEDF could inhibit BLM-induced inflammatory response. Figure S4. Delayed intervention of PEDF could also inhibit collagen deposition. Figure S5. PEDF expression level in RPL fibroblasts. [file 12931_2022_2027_MOESM1_ESM.pdf]

Supplementary materials

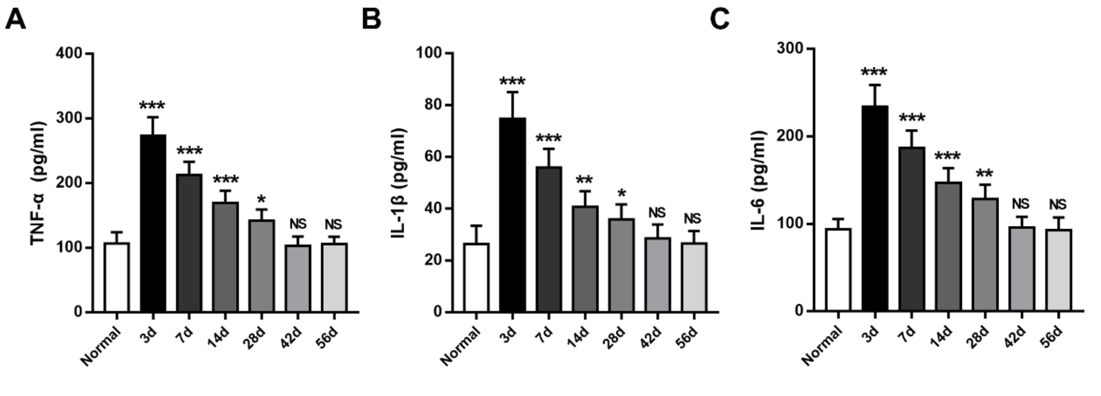

Figure S1. Quantification of cytokines. (A) TNF-α, (B) IL-1β, and (C) IL-6 expression levels increased in the BLM-treated groups, and the highest expression was observed in the 3d group. Data are expressed as the mean ± SD. n = 6. NS, no significant difference; *$P < 0.05$; **$P < 0.01$; ***$P < 0.001$.

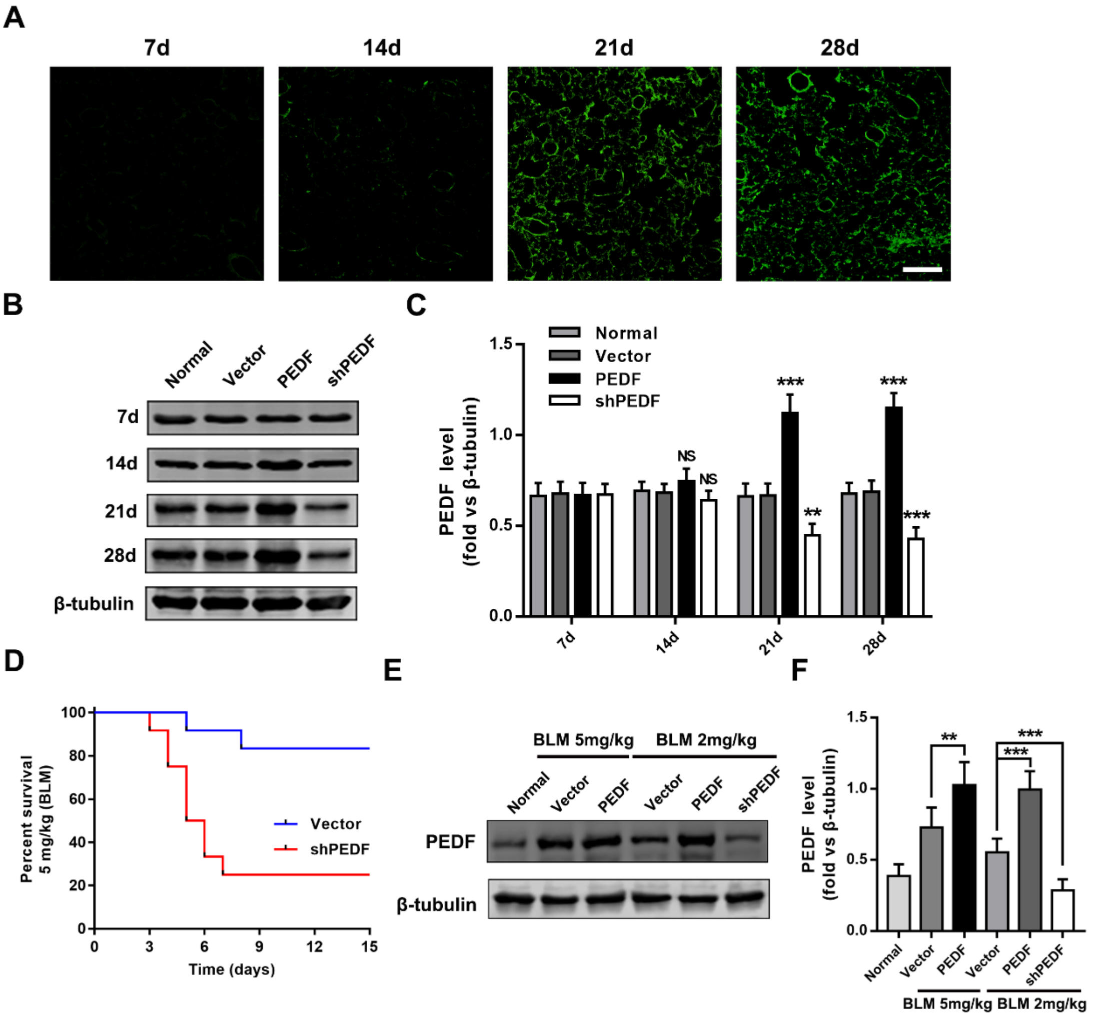

Figure S2. PEDF expression level in rats. (A) Representative GFP expression images were shown. Both the blank AAVs and PEDF-AAVs were constructed and labeled with GFP. The lungs were harvested from referential time point. bar=200μm. (B) and (C) Western blot to detect the transfection efficiency of AAVs (PEDF-AAV or PEDF-siRNA-AAV). Data are expressed as the mean ± SD, n = 6. (D) The survival curve of shPEDF rats after exposure to BLM (5 mg/kg). n=12. (E) and (F) 28 days after BLM-exposed, western blot to detect the transfection efficiency of AAVs (PEDF-AAV or PEDF-siRNA-AAV). n=6. NS, no significant difference; **P < 0.01; ***P < 0.001.

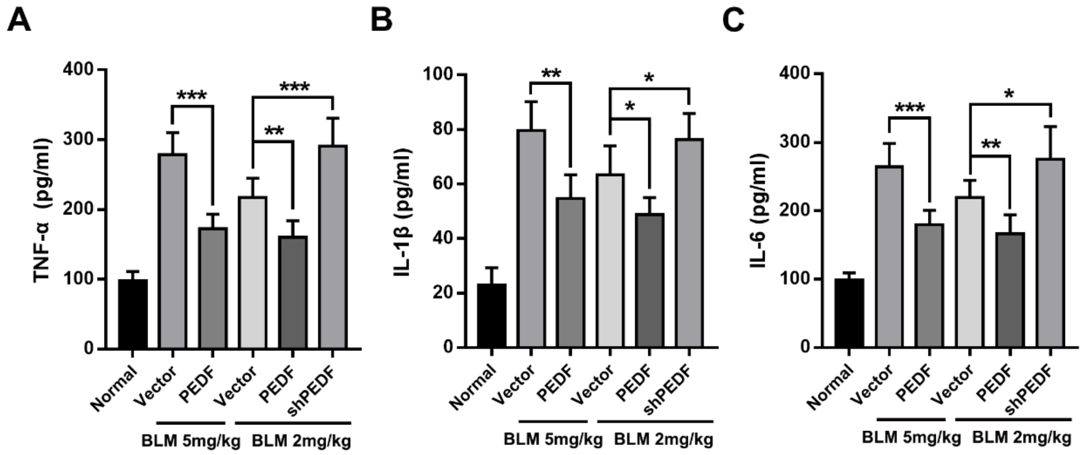

Figure S3. PEDF could inhibit BLM-induced inflammatory response. Quantification of (A) TNF-α, (B) IL-1β, and (C) IL-6 expression levels of the BALF from the experimental group and AAV treatment group (3 days after BLM-exposed). Data are expressed as the mean ± SD, n = 6, *P < 0.05; **P < 0.01; ***P < 0.001.

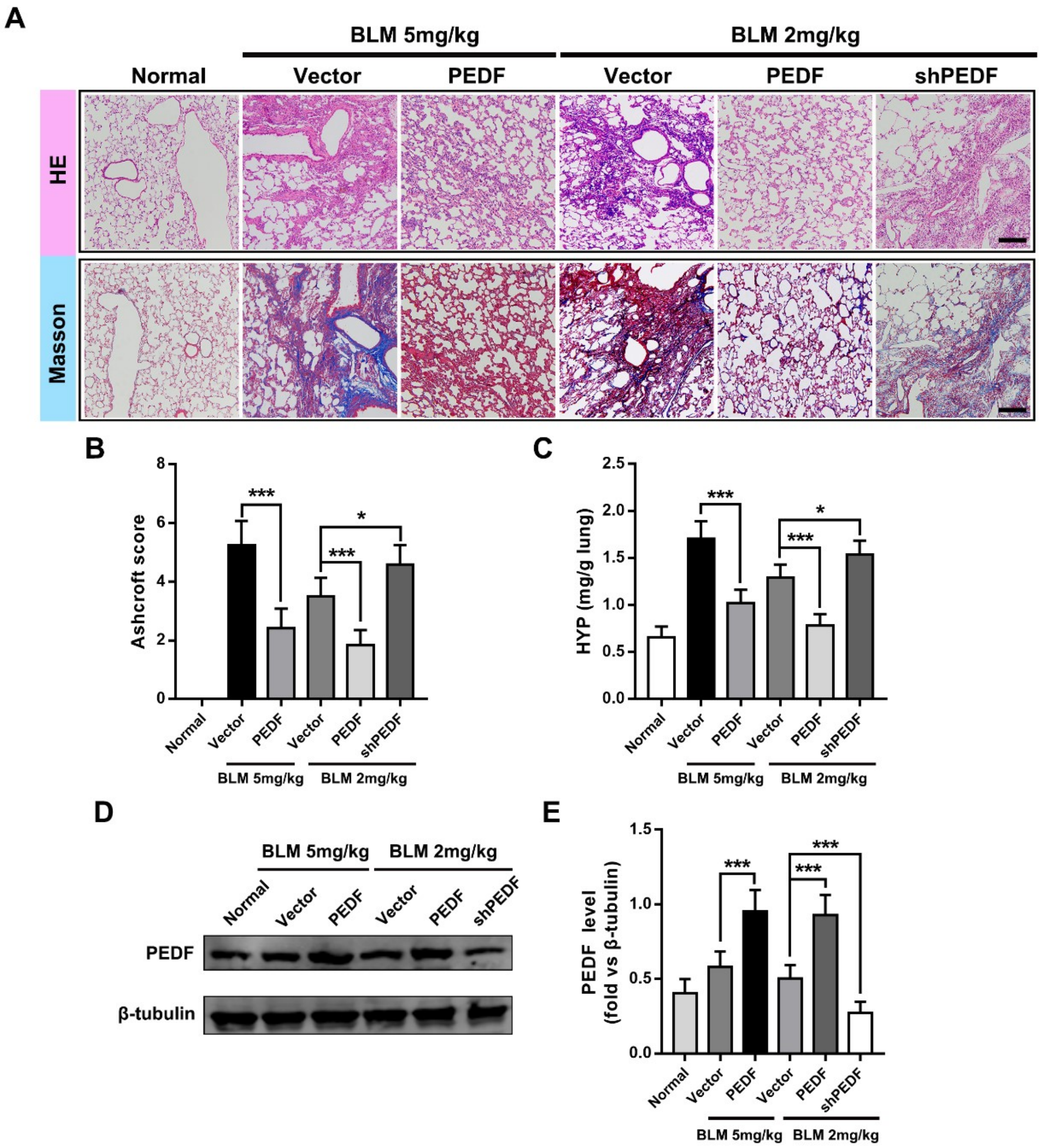

Figure S4. Delayed intervention of PEDF could also inhibit collagen deposition. (A) Representative images of the H&E and Masson-stained cross-section of the lung from the experimental group and delayed treatment group (42 days after BLM-exposed). Bar = 200μm. (B) The Ashcroft score among multiple groups. (C) Quantification of hydroxyproline content in lung tissue of each group. Data are expressed as the mean ± SD, n = 6, *P < 0.05; ***P < 0.001. (D) and (E) 42 days after BLM-exposed, western blot to detect the transfection efficiency of AAVs (PEDF-AAV or PEDF-siRNA-AAV). n=6. **P < 0.01; ***P < 0.001.

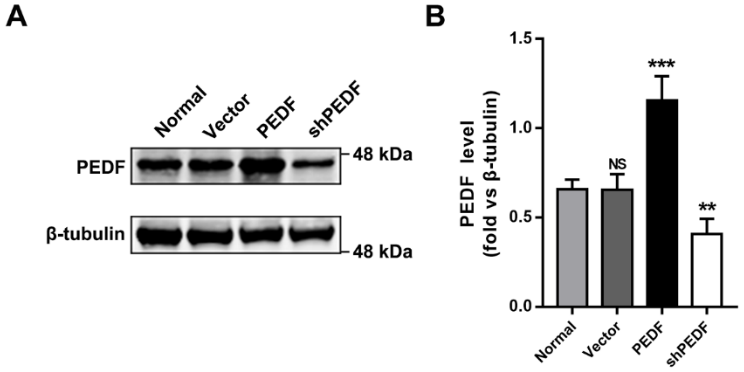

Figure S5. PEDF expression level in RPL fibroblasts. (A) and (B) Western blot to detect the transfection efficiency of LVs (PEDF-LV or PEDF-siRNA-LV). Data are expressed as the mean ± SD, n = 4, NS, no significant difference; $^{**}P < 0.01$; $^{***}P < 0.001$.
